# Assessment of Potential Zoonotic Disease Exposure and Illness Related to an Annual Bat Festival — Idanre, Nigeria

**Published:** 2014-04-18

**Authors:** Neil M. Vora, Modupe Osinubi, Ryan M. Wallace, Abimbola Aman-Oloniyo, Yemi H. Gbadegesin, Yennan Kerecvel Sebastian, Olugbon Abdullateef Saliman, Mike Niezgoda, Lora Davis, Sergio Recuenco

**Affiliations:** 1 EIS officer, CDC; 2 Division of High-Consequence Pathogens and Pathology, National Center for Emerging and Zoonotic Infectious Diseases, CDC; 3 Field Epidemiology and Laboratory Training Program, Abuja, Nigeria; 4 Federal Ministry of Health, Abuja, Nigeria; 5 Global Immunization Division, Center for Global Health, CDC

Bats provide vital ecologic services that humans benefit from, such as seed dispersal and pest control, and are a food source for some human populations. However, bats also are reservoirs for a number of high-consequence zoonoses, including paramyxoviruses, filoviruses, and lyssaviruses (1). The variety of viruses that bats harbor might be related to their evolutionary diversity, ability to fly large distances, long lifespans, and gregarious roosting behaviors (1,2). Every year a festival takes place in Idanre, Nigeria, in which males of all ages enter designated caves to capture bats; persons are forbidden from entering the caves outside of these festivities. Festival participants use a variety of techniques to capture bats, but protective equipment rarely is used, placing hunters at risk for bat scratches and bites. Many captured bats are prepared as food, but some are transported to markets in other parts of the country for sale as bushmeat. Bats also are presented to dignitaries in elaborate rituals. The health consequences of contact with these bats are unknown, but a number of viruses have been previously identified among Nigerian bats, including lyssaviruses, pegiviruses, and coronaviruses (2–4). Furthermore, the caves are home to *Rousettus aegyptiacus* bats, which are reservoirs for Marburg virus in other parts of Africa (5).

In February 2013, a team composed of members of the Nigerian Field Epidemiology and Laboratory Training Program (FELTP), the Nigerian Federal Ministry of Health, and CDC traveled to Idanre to assess potential zoonotic disease exposures and illnesses related to the festival. Interviews conducted with 54 persons who have participated in the festival as bat hunters revealed that 43 (80%) had a history of bat scratches and 39 (72%) had a history of bat bites. Only one (1.9%) hunter reported ever having received rabies vaccine. None of the hunters knew of a person who had acquired a fatal illness as a result of contact with bats or entering the caves. Additional data analyses and serologic assays are pending.

Driven by socioeconomic and environmental factors, the emergence of infectious diseases has accelerated in recent years. Most emerging infectious diseases are zoonotic, and many have wildlife origins (1,6). Investigations of newly identified infectious diseases, such as severe acute respiratory syndrome (SARS) and Nipah virus infection, have historically been reactive, requiring the sudden application of resources to the investigation and control of an outbreak. A proactive approach involving enhanced scientific and surveillance efforts in areas identified as emerging infectious disease “hotspots” during periods when there is no known epidemic might improve the detection of novel pathogens or recognition of outbreaks.

Through programs such as the Nigerian FELTP, the epidemiologic and laboratory resources needed to identify pathogens and outbreaks are now reaching areas of the world where resources have previously been limited. The investigation in Idanre is an example of an FELTP investigation of an activity that puts persons at risk for pathogen exposure. Particular topics for further evaluation include the factors that promote pathogen transmission from bats to humans, such as habitat encroachment and trade in bushmeat (1). Public health interventions to improve access to rabies vaccine and personal protective equipment for persons at risk for bat exposures are likely to be beneficial.
